# Response to: Comment on “Management of Atrial Fibrillation in Critically Ill Patients”

**DOI:** 10.1155/2016/9724504

**Published:** 2016-08-25

**Authors:** Mattia Arrigo, Dominique Bettex, Alain Rudiger

**Affiliations:** University Heart Center, University Hospital Zurich, 8091 Zürich, Switzerland

We kindly disagree with the comments of Dr. Jelliffe on the use of digoxin in critically ill patients with atrial fibrillation (AF) based on the review article “Management of Atrial Fibrillation in Critically Ill Patients” [1].

Our daily experience in treating severely ill patients with new-onset AF shows that digoxin is helpful in reducing the heart rate (rate-control strategy) but is not useful for converting AF into sinus rhythm (rhythm-control strategy). This is in line with the current guidelines of the European Society of Cardiology [2] that state that “digoxin is ineffective for AF termination.” Based on multiple negative randomized placebo-controlled studies, the guidelines give a class III A recommendation for converting new-onset AF with digoxin. The 4 patients reported by Roger Jelliffe have, though interesting, only anecdotal value.

Furthermore, Dr. Jelliffe disagrees with our statement that serum levels of digoxin are recommended to support the diagnosis of intoxication but are not very helpful for titrating dose, as plasma levels do not correlate well with clinical response. He suggests measurements of plasma levels of digoxin combined with the use of the BestDose software to calculate the optimal regimen. However, in his publication Dr. Jelliffe states that “*the range of serum digoxin concentrations generally regarded as being therapeutic is often said to be from about 0.5 to 2.0 ng/mL (…) a few patients tolerate serum digoxin concentrations well over 3 ng/mL, and actually as high as 6.5 ng/mL, without any toxicity (…) patients with AF might require their own separate therapeutic ranges or target concentrations.*”

This further supports our statement that digoxin should be titrated based on clinical response and not aiming at a predefined serum concentration. The reported method for adjusting the dose of digoxin using the BestDose software is interesting but its broad implementation in daily clinical practice in ICUs is unfortunately not in sight.
